# Coronary Artery Disease and Symptomatic Severe Aortic Valve Stenosis: Clinical Outcomes after Transcatheter Aortic Valve Implantation

**DOI:** 10.3389/fcvm.2015.00018

**Published:** 2015-04-15

**Authors:** Jennifer Mancio, Ricardo Fontes-Carvalho, Marco Oliveira, Daniel Caeiro, Pedro Braga, Nuno Bettencourt, Vasco Gama Ribeiro

**Affiliations:** ^1^Department of Cardiology, Gaia Hospital Center, Vila Nova de Gaia, Portugal; ^2^Cardiovascular R&D Unit, Department of Physiology and Cardiothoracic Surgery, Faculty of Medicine of Porto University, Porto, Portugal

**Keywords:** coronary disease, aortic valve stenosis, transcatheter aortic valve implantation, percutaneous coronary angioplasty intervention, prognosis

## Abstract

**Introduction:**

The impact of coronary artery disease (CAD) on outcomes after transcatheter aortic valve implantation (TAVI) has not been clarified. Furthermore, less is known about the indication and strategy of revascularization in these high risk patients.

**Aims:**

This study sought to determine the prevalence and prognostic impact of CAD in patients undergoing TAVI, and to assess the safety and feasibility of percutaneous coronary intervention (PCI) before TAVI.

**Methods:**

Patients with severe aortic stenosis (AS) undergoing TAVI were included into a prospective single center registry from 2007 to 2012. Clinical outcomes were compared between patients with and without CAD. In some patients with CAD, it was decided to perform elective PCI before TAVI after decision by the Heart team. The primary endpoints were 30-day and 2-year all-cause mortality.

**Results:**

A total of 91 consecutive patients with mean age of 79 ± 9 years (52% men) underwent TAVI with a median follow-up duration of 16 months (interquartile range of 27.6 months). CAD was present on 46 patients (51%). At 30-day, the incidences of death were similar between CAD and non-CAD patients (9 and 5%, *p* = 0.44), but at 2 years were 50% in CAD patients and 24% in non-CAD patients [crude hazard ratio with CAD, 2.2; 95% confidence interval (CI), 1.1–4.6; *p* = 0.04]. Adjusting for age, gender, left ventricular ejection fraction, and glomerular filtration rate, the hazard of death was 2.6-fold higher in patients with CAD (95% CI, 1.1–6.0; *p* = 0.03). Elective PCI before TAVI was performed in 13 patients (28% of CAD patients). There were no more adverse events in patients who underwent TAVI + PCI when compared with those who underwent isolated TAVI.

**Conclusion:**

In severe symptomatic AS who underwent TAVI, CAD is frequent and adversely impacts long-term outcomes, but not procedure outcomes. In selected patients, PCI before TAVI appears to be feasible and safe.

## Introduction

Coronary artery disease (CAD) affects 25–50% of patients with severe aortic stenosis (AS) who undergo surgical aortic valve replacement (SAVR). (1) Its presence negatively impacts the periprocedural and long-term outcomes after SAVR. However, improved long-term survival has been reported in patients with AS and CAD who underwent combined SAVR and CABG compared with those receiving isolated SAVR (2). Based on these findings, current guidelines recommend bypass surgery to all significant stenosis at the time of SAVR (3, 4).

The prevalence and impact of CAD on outcomes after transcatheter aortic valve implantation (TAVI) have not been fully delineated. Published studies specifically addressing this issue are sparse and controversial (5–8). Moreover, the only randomized clinical trial that compares SAVR with TAVI excluded patients with CAD requiring revascularization (9). Consequently, the original TAVI protocol precludes that significant CAD must be treated by percutaneous coronary intervention (PCI) before TAVI, and that CAD not treatable by PCI should be considered a formal contraindication for TAVI (4, 10). However, it is unknown if revascularization in the high-risk population referred to TAVI is associated with similar benefits to those observed in patient referred to SAVR. Furthermore, performing PCI in a patient with severe AS can be problematic; thus, the risks of PCI in the setting of AS are unknown (11). Since CABG is the primary mode of revascularization in patients with CAD and moderate to severe AS, PCI has been limited to patients with AS who presented with acute coronary syndrome. Furthermore, the safety of TAVI has just been investigated isolated from concomitant revascularization procedures (12, 13).

The aims of the present study were to determine the prevalence of CAD and its impact on procedural outcomes and long-term survival in patients undergoing TAVI, and to assess the safety and feasibility of PCI before TAVI.

## Materials and Methods

### Patient population

Consecutive patients with symptomatic AS undergoing TAVI at Centro Hospitalar de Vila Nova de Gaia were enrolled in a prospective registry from August 2007 to October 2012. The study complied with the declaration of Helsinki, and the registry was approved by the local ethics committee. All the patients provided written informed consent to participate in our registry with prospective follow-up assessment.

Initial assessment included clinical evaluation by the Heart Team, transthoracic and transesophageal echocardiography, invasive coronary angiography, and computed tomography of the aorta, iliac, and common femoral arteries. In all patients, the logistic European System for Cardiac Operative Risk Evaluation (EuroSCORE), EuroSCORE 2 (14), and the Society of Thoracic Surgeons Predicted Risk of Mortality (STS-PROM) (15) were calculated. Patients were excluded from the analysis if the procedure was considered unsuccessful according to criteria in the Valve Academic Research Consortium (VARC-I) guidelines (16). TAVI was performed using the Medtronic CoreValve system (Medtronic, Minneapolis, MN, USA) or the Edwards SAPIEN valve (Edwards Lifesciences, Irvine, CA, USA) by transfemoral, transapical, or transsubclavian approach according to the instructions for use and as previously described (9, 12). Devices and access site were selected by anatomical and technical features. In some patients, it was decided to perform elective PCI before TAVI either in a planned intervention prior to TAVI (staged PCI) or at the time of TAVI (concomitant PCI). In cases of concomitant PCI, patients first underwent PCI followed by TAVI in the same session. Revascularization strategy was based on the amount of myocardium at risk, which was estimated integrating the visual assessment of the coronary anatomy, the presence of wall motion alteration in the echocardiogram, and history of prior myocardial infarction. On the day before TAVI, patients undergoing isolated TAVI were medicated with 250 mg of aspirin and 300 mg of clopidogrel, and the patients undergoing concomitant PCI were medicated with 250 mg of acetylsalicylic acid and 600 mg of clopidogrel. Procedural anticoagulation was obtained with a heparin bolus of 70 IU/kg, aiming for an activated clotting time of 250–300 ms.

### Angiographic analysis

For the purpose of this study, baseline coronary angiographies were analyzed by two experienced interventional cardiologists. CAD group included patients with at least one lesion on the epicardial coronary arteries with ≥50% diameter stenosis or patients who had been submitted to coronary revascularization by PCI or CABG before AS diagnosis.

### Study end points and definitions

Primary end points included 30-day and 2-year all-cause mortality. Prespecified secondary end points included mortality from cardiovascular causes, myocardial infarction, stroke, acute renal injury, vascular complications, bleeding, new onset atrial fibrillation, and pacemaker implantation. In a retrospective analysis of neurologic events, major stroke was defined by a score of at least 2 on the modified Rankin scale (which ranges from 0 to 6, with higher scores indicating greater disability). All events were adjudicated according to the VARC I definitions (16) by a clinical event committee consisting of invasive cardiologists and cardiac surgeons. Patients were followed during the index hospitalization and at 30 days, 6 months, 1 year, and yearly thereafter.

### Statistical analysis

Patients were stratified into two groups according to the presence or absence of CAD. Baseline characteristics and clinical outcomes were compared between groups. Continuous variables were described using the mean ± SD if normally distributed or median and interquartile range (IQR) when non-normally distributed. Student’s *t*-test was used for the comparisons between continuous variables if normally distributed, and Mann–Whitney *U* in cases of non-normally distributed variables. Categorical variables were described using relative frequencies, and compared between CAD and non-CAD patients using the Chi-square test or Fisher exact test when appropriate. Clinical outcomes at 30 days and 2 years were expressed as counts or incidence rates computed according to Kaplan–Meier analysis. Hazard ratios were derived from Mantel-Cox log-rank for all-cause mortality. Clinical outcomes at 30 days and 2 years were also compared between patients with and without CAD. A Cox multivariate regression analysis was performed to determine adjusted hazard ratio of death between groups. We estimated that a sample size of 91 patients (controls ratio of 0.49) allowed the detection of an hazard ratio difference of 0.5 for the primary end point with a type II error of 20% and a significance level of 5% (two-sample comparison of survivor functions log-rank test by the Freedman method). To assess feasibility and safety of PCI before TAVI, the clinical outcomes at 30 days were compared between patients with CAD who underwent isolated TAVI and TAVI + PCI. A *p*-value <0.05 was considered to indicate a statistically significant difference. Statistical analysis was performed using SPSS 19.0 for Windows (SPSS, Inc., Chicago, IL, USA).

## Results

**Figure 1:**
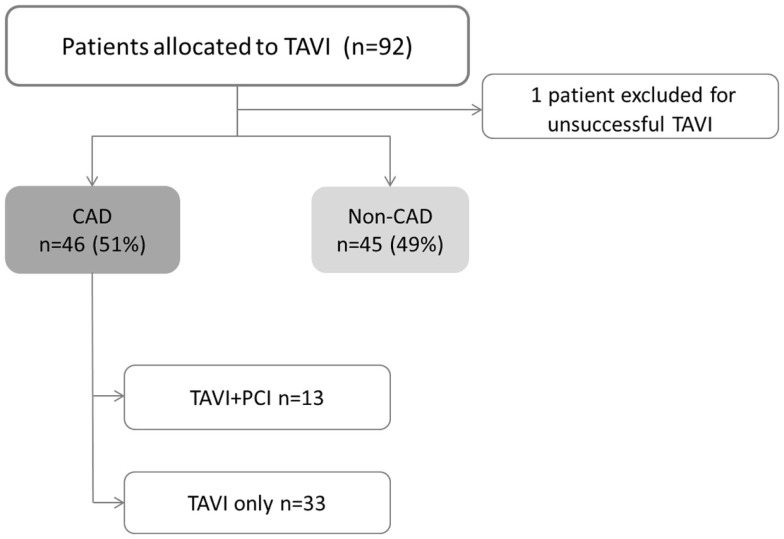
**Study flowchart**. CAD, coronary artery disease; PCI, percutaneous coronary intervention; TAVI, transcatheter aortic valve implantation. TAVI + PCI combined procedure including both TAVI and PCI.

A total of 91 consecutive patients (52% men) with mean age of 79 ± 9 years underwent TAVI. CAD was present on 46 patients (51%). Among CAD group, 26 patients (48%) had prior revascularization, 8 patients (17%) had “*de novo*” significant CAD, and 13 patients (28%) needed elective PCI before TAVI (Figure 1). Scheduled follow-up was completed in all patients with a median duration of 16 months (IQR of 27.6 months, Quartile 25 of 5.6 months).

### Baseline characteristics

Baseline clinical and echocardiographic characteristics are summarized in Table 1. Patients with CAD were more frequently men (67 and 33%, *p* < 0.01), hypertensive (89 and 67%, *p* = 0.01), dyslipidemic (76 and 53%, *p* = 0.02), had worse renal function, [glomerular filtration rate (GFR): 50 ± 19 and 64 ± 33 mL/min/m^2^, *p* = 0.02], and left ventricular systolic function (left ventricular ejection fraction, LVEF: 47 ± 10 and 53 ± 9%, *p* < 0.01 with a prevalence of LV dysfunction in CAD of 46% and in non-CAD of 20%, *p* = 0.021). Mean EuroScore 2 was significantly higher in CAD group (9 ± 4 and 5 ± 4, *p* < 0.01). Patients with CAD had similar age, aortic valve area, and functional class of heart failure when compared with those without CAD.

**Table 1:** **Comparison of baseline clinical, echocardiographic, and procedural characteristics between CAD and non-CAD groups**.

	All (*n* = 91)	CAD groups		*p*-Value
		CAD (*n* = 46)	Non-CAD (*n* = 45)	
Age (years)	79 ± 9	79 ± 7	78 ± 7	0.283
Male gender, *n* (%)	47 (52)	32 (67)	15 (33)	0.001
STS-PROM score	6 ± 5	7 ± 5	6 ± 5	0.559
EuroScore 2	7 ± 5	9 ± 4	5 ± 4	0.004
Hypertension, *n* (%)	71 (78)	41 (89)	30 (67)	0.010
Dyslipidemia, *n* (%)	59 (65)	35 (76)	24 (53)	0.023
Diabetes, *n* (%)	35 (38)	19 (41)	16 (36)	0.573
Class III or IV NYHA, *n* (%)	61 (67)	30 (65)	31 (69)	0.710
Prior PCI, *n* (%)	8 (9)	8 (17)		
Prior CABG, *n* (%)	16 (19)	16 (35)		
CVD, *n* (%)	21 (23)	13 (28)	8 (18)	0.235
PAD, *n* (%)	35 (17)	21 (46)	14 (31)	0.154
COPD, *n* (%)	39 (43)	17 (37)	22 (49)	0.250
GFR (mL/min/m^2^)	58 ± 28	50 ± 19	64 ± 33	0.012
Renal dysfunction, *n* (%)	60 (66)	37 (82)	23 (50)	0.008
AF, *n* (%)	29 (32)	15 (33)	14 (31)	0.878
Permanent pacemaker, *n* (%)	7 (8)	3 (7)	4 (9)	0.672
*Echocardiografic evaluation*
AVA, cm^2^	0.6 ± 0.1	0.6 ± 0.2	0.6 ± 0.1	0.850
Mean AV gradient (mmHg)	51 ± 13	50 ± 13	52 ± 13	0.505
LVEF (%)	50 ± 10	47 ± 10	53 ± 9	0.006
LV dysfunction^a^, *n* (%)	30 (33)	21 (46)	9 (20)	0.0210
Moderate or severe MR, *n* (%)	5 (7)	1 (2)	4 (9)	0.125
*Procedure features*				
Transarterial access, *n* (%)	87 (96)	44 (96)	43 (96)	0.982
Transapical access, *n* (%)	4 (4)	2 (4)	2 (4)	0.999
Medtronic/coreValve, *n* (%)	79 (87)	38 (83)	41 (91)	0.231
Edwards SAPIEN, *n* (%)	12 (13)	8 (17)	4 (9)	0.072

*Results are presented as mean ± SD*.

**p*-Values shown compare CAD and non-CAD groups*.

*AF, atrial fibrillation; AV, aortic valve; AVA, aortic valve area; CABG, coronary arteries bypass graft; COPD chronic obstructive pulmonary disease; CVD cerebrovascular disease; GFR, glomerular filtration rate; LVEF, left ventricular ejection fraction; MR, mitral regurgitation; NYHA, New York Heart Association; PAD, peripheral artery disease; PCI, percutaneous coronary intervention; STS, Society of Thoracic Surgeons*.

*^a^Left ventricular systolic dysfunction was defined as ejection fraction lower than 50%*.

### Clinical outcomes

**Figure 2:**
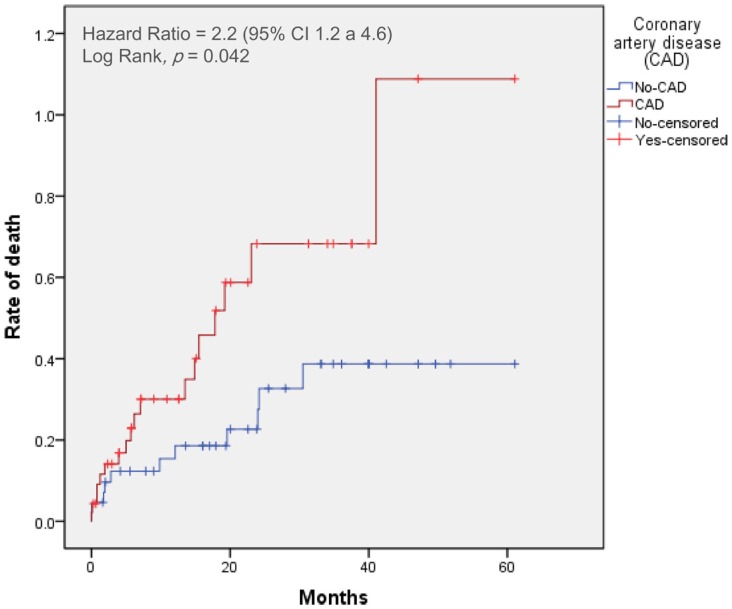
**Time-to-event curves for the primary end point**. Events were calculated with the use of Kaplan–Meier methods and compared with the use of a log-rank test. CAD, coronary artery disease; TAVI, transcatheter aortic valve implantation.

Clinical outcomes at 30 days and 2 years after TAVI with or without CAD are presented in Table 2. Time-to-event curves for the primary end point are displayed in Figure 2.

**Table 2:** **Clinical outcomes at 30 days and 2 years with or without CAD**.

Outcome	30 days		*p*–Value*	2 years		*p*-Value*
	CAD *n* = 46	Non-CAD *n* = 45		CAD *n* = 46	Non-CAD *n* = 45	
	No of patients (%)			No of patients (%)	
Death from all causes	4 (9)	2 (5)	0.446	17 (50)	9 (24)	0.042
Death from cardiovascular causes	2 (3)	1 (3)	0.890	10 (35)	3 (8)	0.038
Stroke or TIA	5 (11)	3 (7)	0.520	9 (20)	5 (15)	0.321
Myocardial infarction	0	0		1 (2)	0	0.160
Major vascular complications	5 (11)	4 (10)	0.441	6 (12)	4 (10)	0.448
Major bleeding	5 (11)	4 (10)	0.441	6 (12)	4 (10)	0.448
Acute renal lesion	6 (13)	4 (10)	0.580	7 (14)	5 (11)	0.652
NOAF	11 (30)	8 (20)	0.621	11 (30)	9 (23)	0.601
New pacemaker implantation	16 (34)	12 (27)	0.442	16 (34)	12 (27)	0.458

*All percentages are Kaplan–Meier estimates at the specific time point and thus do not equal the number of patients divided by the total of number in the study group. CAD, coronary artery disease; NOAF, new onset atrial fibrillation; TIA, transient ischemic attack*.

*p-Values are for the between-group comparison of the frequency of the event at each time point.

At 30 days, the primary end point was not statistically different between patients with and without CAD (incidence of mortality with CAD: 9%; non-CAD: 5%, *p* = 0.44). The 30-day major stroke (CAD: 5%; non-CAD: 2%, *p* = 0.52), life-threatening bleeding (CAD: 18%; non-CAD: 15%, *p* = 0.32), and major vascular complications (CAD: 10%; non-CAD: 11%, *p* = 0.44) were not statistically different when comparing CAD and non-CAD groups.

The 2-year-mortality was 50% in CAD patients and 24% in non-CAD patients (crude hazard ratio with CAD, 2.2; 95% confidence interval [CI], 1.1–4.6; *p* = 0.04). Adjusting for age, gender, hypertension, dyslipidemia, LVEF, and GFR, the hazard of death was 2.6-fold higher in patients with CAD (95% CI, 1.1–6.0; *p* = 0.03). Mortality from cardiovascular causes was also significantly higher in patients with CAD (hazard ratio with CAD of 2.2; 95% CI from 1.2 to 4.6, *p* = 0.04). No differences were found in cumulative incidence of the others secondary end points (Table 2).

### Feasibility and safety of PCI before TAVI

**Figure 3:**
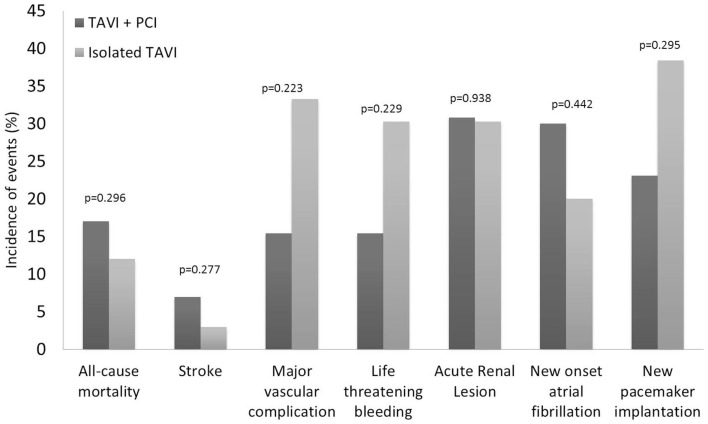
**Clinical outcomes at 30 days according to the Valve Academic Research Consortium (VARC) safety endpoints for patients who underwent isolated TAVI and elective PCI before TAVI**. PCI, percutaneous coronary intervention; TAVI, transcatheter aortic valve implantation.

Procedural characteristics of PCI before TAVI were described in Table 3. Elective PCI before TAVI was performed in 13 patients: 11 in a planned intervention (*staged PCI*) in a median of 56 days before TAVI, and 2 at the time of TAVI (*concomitant PCI*). The most frequent (5 of 13 cases) PCI concerned patients with severe stenosis of vein graft. Drug eluting stents were used in the majority of cases (9 of 13 cases). There were no major adverse events after PCI. Thirty-day all-cause mortality was similar between patients treated with TAVI + PCI (17%) or isolated TAVI (10%) (*p* = 0.296). Incidence of 30-day major stroke (*p* = 0.270), life-threatening bleeding (*p* = 0.229), and major vascular complications (*p* = 0.229) did not differ between patients who underwent TAVI + PCI or isolated TAVI (Figure 3).

**Table 3:** **Procedural characteristics of elective PCI before TAVI**.

	TAVI + PCI *n* = 13
Time to TAVI – days^a^	40 (0–166)
Concomitant procedure, *n* (%)	2 (15)
Staged procedures, *n* (%)	11 (82)
Time to TAVI – days^a^	56 (3–166)
Vessel, *n* (%)	
LM	3 (23)
LAD	4 (30)
LCX	1 (8)
RCA	0
Vein graft	5 (39)
Stents per patient, *n* (%)	
1	11 (84)
2	2 (15)
Drug eluting stents, *n* (%)	9 (75)

*^a^Results are presented as median (range)*.

*LAD, left descending artery; LM, left main, LCX, left circumflex artery; PCI, percutaneous coronary intervention; RCA, right coronary artery; TAVI, transcatheter aortic valve implantation. TAVI + PCI combined procedure including both PCI and TAVI*.

## Discussion

This study showed that significant CAD is very frequent in patients undergoing TAVI implantation and its presence adversely impacts long-term outcomes, but not periprocedural outcomes. In selected patients, PCI before TAVI appears to be feasible and safe.

### CAD is frequent in patients undergoing TAVI

In our study, CAD was observed in more than half of patients who underwent TAVI. The prevalence of CAD reported in the few publications on this topic ranges from 49 to 75% (5–8). Various definitions of CAD had been used on the available literature which could account, in part, for the differences in results. Masson et al. (6) used a broad definition of CAD including patients with prior revascularization or any coronary lesion of at least 50% in angiography, and as expected, they reported the higher prevalence of co-existent CAD (6). Dewel et al. (5) defined CAD as prior coronary revascularization excluding those with significant CAD treated medically. Despite the wide variety of CAD definition, it seems common that in patients referred for TAVI, the prevalence of CAD tends to be higher than SAVR series of AS patients. This can be explained in part as consequence of selection bias (i.e., older patients or previous CABG tend to be more frequently refused for surgery and to undergo TAVI as an alternative procedure).

### CAD management in the setting of TAVI

The current 2014 guidelines for myocardial revascularization in patients with stable CAD recommend to perform revascularization only in cases with documented ischemia by non-invasive imaging stress tests or invasive fractional flow reserve assessment (FFR) <0.8 (17). However, in patients with symptomatic severe AS and concomitant CAD, it remains indicated to revascularize all the significant coronary stenosis visualized on the angiography by CABG at the same time of SAVR (4), or by PCI at least 1 month before TAVI (4, 10).

#### Detection of ischemia in patients with AS

In stable angina, Boden et al. showed that revascularization should not be routinely performed in the presence of significant coronary stenosis on angiography even in symptomatic patients (18). Furthermore, FAME trials clearly demonstrate that diameter stenosis is not a good marker of significance of disease and that revascularization should be guided according to functional evaluation. (19) Currently, it is possible invasively and non-invasively to identify myocardial ischemia; however, there remains many concerns about the safety and diagnostic accuracy of cardiac stress tests in patients with symptomatic severe AS.

A protocol of 6-min perfusion adenosine to assess myocardium perfusion by SPECT in patients with severe AS showed to be safe and diagnostically accurate for the presence of CAD (20). Recently, dobutamine stress echocardiogram was helpful to assess severity and to predict outcomes in patients with paradoxical low-flow gradient AS with preserved ejection fraction referred to TAVI with no major adverse effects. (21). Despite these data in favor of stress tests safety in severe AS, its diagnostic value is unknown. Typically, coronary artery flow reserve is impaired in AS, which may affect the FFR cut-off reference value. (22) Moreover, in patients with AS, left ventricular hypertrophy may be accompanied by interstitial myocardial fibrosis starting at the subendocardial layers and progressing toward replacement fibrosis. (23) These alterations gradually affect left ventricular systolic and diastolic function, which can lead to segmental kinetic alterations and myocardial perfusion defects misclassifications in stress echocardiogram and cardiac magnetic resonance imaging (CMRI).

#### Impact of ischemia on periprocedural and long-term outcomes after TAVI

The rationale of revascularization before TAVI is based on the belief that worsening of myocardial ischemia during rapid ventricular pacing could occur in the presence of non-revascularized CAD. However, since TAVI is a less invasive procedure than surgical replacement, the procedure advantages of revascularization may be more discrete than in SAVR. Furthermore, beyond CAD, various hypotheses exist on the underlying mechanisms of periprocedural myocardial injury in patients undergoing TAVI. These include global myocardial ischemia due to extreme hypotension, direct trauma during balloon inflation or prosthesis placement, and coronary embolization of aortic valve debris. In a recent study, Kim et al. determined the incidence and degree of ischemic myocardial damage using CMRI and myocardial biomarkers in patients undergoing TAVI; these authors found that new late myocardium gadolinium enhancement (LGE) after TAVI was observed in a notable proportion of patients (18%), but the majorly was small size, subendocardial, or intramural, and multifocal suggesting embolic origin. Interestingly, no correlation was found between pre-existing coronary stenosis and the localization of new infarctions (24). In our study, CAD did not associate with higher risk of 30-day mortality. Our results are closer to those of Masson et al. (6), who observed similar 30-day mortality in patients with and without CAD (11.5 vs. 6.3%, *p* = 0.39), and those of Gautier et al. (7), who did not demonstrate influence of CAD on 30-day mortality after TAVI. In The PARTNER US Trial, a subgroup analysis did not show any significant interaction between the presence of prior coronary revascularization and the effect of TAVI treatment on 30-day mortality (9, 12). Consistent with these results, our findings suggest that CAD has limited impact on the periprocedural risk of TAVI. Contrary, Dewey et al. (5) showed a 30-day mortality 10-fold higher among CAD compared to non-CAD patients (13.1 vs. 1.2%, *p* = 0.002). In this study, CAD group only included patients with previous PCI or CABG. This restrictive CAD definition could justify the higher mean STS predicted risk of mortality (13 ± 4) of this population when compared with our CAD patients (7 ± 5).

Few studies are available on the long-term effect of CAD on TAVI outcomes. Congruent with our findings, incomplete revascularization was demonstrated to be an independent predictor of decreased left ventricular recovery and was associated with higher 1-year mortality. (25) A comparable result was found when the SYNTAX score was applied for the evaluation of the revascularization status, but not when significant CAD was defined using a visual estimation alone. (26) By contrast, a revascularization strategy selection by a dedicated Heart Team was reported to provide a similar long-term prognosis independently of the revascularization status; the presence of incomplete coronary revascularization did not worsen the 1-year survival (27). Similarly, Masson et al. (6) showed no impact of CAD on 1-year mortality after TAVI. In the Multicentre Canadian Experience, coronary status was not predictor of cumulative late mortality after TAVI, as well (28). Despite differences between CAD definition and revascularization status, it has been speculated that the benefit of revascularization on survival improvement may be mitigated by a shorter life expectancy and less active lifestyle in the older TAVI population.

#### Safety of PCI in patients undergoing TAVI

Goel and colleagues, in a systematic analysis comparing the outcomes after PCI in 254 patients with severe AS with 508 patients without AS, found that the 30-day mortality was similar between patients with or without AS (11). This finding led the authors to speculate that PCI could be an alternative in the management of CAD in patients considered to TAVI. However, a new pattern of complications must be considered when performing PCI and TAVI (28–31). PCI before TAVI may increase bleeding and vascular complications due to dual antiplatelet therapy and could also increase the risk of stroke and contrast-related kidney injury in this elderly co-morbid group of patients. Data on safety and feasibility of the strategy of PCI before TAVI are limited to a single-center encouraging experience (29, 30). Abdel-Wahab M et al. evaluated the 30-day and 6-month safety of PCI before TAVI; the VARC-defined combined safety end point (11vs. 13%, *p* = 0.74) did not differ between patients submitted to TAVI + PCI or isolated TAVI (29).

### Study limitations

Given the observational design, many limitations must be considered. Our study included several patient subsets (i.e., patients with previously treated CAD, untreated CAD who underwent staged or concomitant PCI) with no information being provided about the completeness of revascularization. The impact of residual myocardial at risk cannot be evaluated. The treatment strategy was based on the Heart Team, taking into consideration an estimative of the myocardial tissue at risk, and the technical complexity. No ischemia and viability imaging tests were performed. Limitations are also due to its monocentric design; however, this aspect gives a clear picture of the impact of a homogeneous treatment strategy.

## Conclusion

In severe symptomatic AS who undergo TAVI, CAD adversely impacts long-term mortality, but not increases the periprocedure risks. In selected patients, PCI before TAVI might be a reasonable revascularization option. Further randomized studies are needed to evaluate the best treatment strategy for CAD, the benefits of revascularization, and the impact of non-revascularized myocardium in outcomes after TAVI. The role of FFR analysis and ischemia imaging tests needs to be explored in this setting.

## Conflict of Interest Statement

The authors declare that the research was conducted in the absence of any commercial or financial relationships that could be construed as a potential conflict of interest.
